# Computed tomography-guided percutaneous cutting needle biopsy for small (≤ 20 mm) lung nodules

**DOI:** 10.1097/MD.0000000000008703

**Published:** 2017-11-17

**Authors:** Guang-Chao Li, Yu-Fei Fu, Wei Cao, Yi-Bing Shi, Tao Wang

**Affiliations:** Department of Radiology, Xuzhou Central Hospital, Xuzhou, Jiangsu, China.

**Keywords:** computed tomography, lung nodule, percutaneous cutting needle biopsy, small

## Abstract

The goal of this study is to determine the feasibility, diagnostic accuracy, and risk factor of complications of computed tomography (CT)-guided percutaneous cutting needle biopsy (PCNB) for small lung nodules.

From January 2014 to May 2015, 141 patients with small lung nodule were performed with CT-guided PCNB procedure. Data on technical success, diagnostic accuracy, and complication were collected and analyzed.

Technical success of CT-guided PCNB for small lung nodules was 100%. A total of 141 nodules were punctured. The mean time of the procedure was 15.7 ± 4.3 minutes. The PCNB results included malignancy (n = 79), suspected malignancy (n = 6), specific benign lesion (n = 8), nonspecific benign lesion (n = 47), and invalid diagnosis (n = 1). The final diagnosis of the 141 nodules included malignancy (n = 90), benign (n = 37), and nondiagnostic lesion (n = 14). The nondiagnostic nodules were not included for calculating the diagnostic accuracy. The sensitivity, specificity, and overall diagnostic accuracy of CT-guided PCNB for small lung nodule were 94.4% (85/90), 100% (37/37), and 96.1% (122/127), respectively. Pneumothorax and lung hemorrhage (≥ grade 2) occurred in 17 (12.1%) and 22 (15.6%) patients, respectively. Based on the univariate and multivariate logistic analyses, the risk factors of pneumothorax included nonprone position (*P* = .019) and longer procedure time (*P* = .018). The independent risk factor of lung hemorrhage (≥ grade 2) was deeper lesion distance from pleura along needle path (*P* = .024).

This study demonstrates that CT-guided PCNB can provide a high diagnostic accuracy for small lung nodule with acceptable complications.

## 1 Introduction

With the wide use of low-dose thoracic computed tomography (CT), more and more lung nodules were detected.^[1–9]^ With the thorough research of the lung nodules, the nodules with the size ≤ 20 mm are called small lung nodules.^[8,9]^ Although the size of the lung nodule is an independent risk factor of malignancy,^[5–7]^ approximately 67.5% to 78% of small lung nodules were malignancy.^[8,9]^ Thus, accuracy pathology diagnosis is a vital step in the management of small lung nodules.

CT-guided fine-needle aspiration biopsy (FNAB) and percutaneous cutting needle biopsy (PCNB) are widely used in the diagnosis of lung nodules.^[9–12]^ FNAB usually shows the cytological features of the lesion, but not the tissue architecture.^[9]^ Furthermore, FNAB has the risk of insufficient tissue sampling.^[9]^ PCNB is a more accurate method of tissue sampling than FNAB because it can obtain enough specimens for pathology diagnosis.^[11,12]^ At present, researches about PCNB for small lung nodules still lack.^[8,9]^

In this study, we determine the feasibility, diagnostic accuracy, and risk factor of complications of CT-guided PCNB for small lung nodules.

## 2 Methods

This single-center, retrospective study was approved by the Institutional Review Board of Xuzhou Central Hospital, and written informed consent was obtained from each participant or their next of kin for procedure and use of their clinical records.

### 2.1 Patients

From January 2014 to May 2015, 141 consecutive patients with small lung nodule were performed with CT-guided PCNB procedure. Baseline data of these 141 patients were demonstrated in Table 1. The inclusion criteria include: newly discovered or enlarging nodule on chest CT, nodule size ≤20 mm. The exclusion criteria include: nodule size <5 mm, patients who required to undergo surgery directly, patients who required follow-up directly. The size of lung nodules was measured along the maximum long-axis diameter. Among the 141 patients, 9 patients had the malignancy history, 20 patients were presented with distant metastases, 2 patients simultaneously had liver mass, and 1 patient simultaneously had ovarial mass.

**Table 1:**
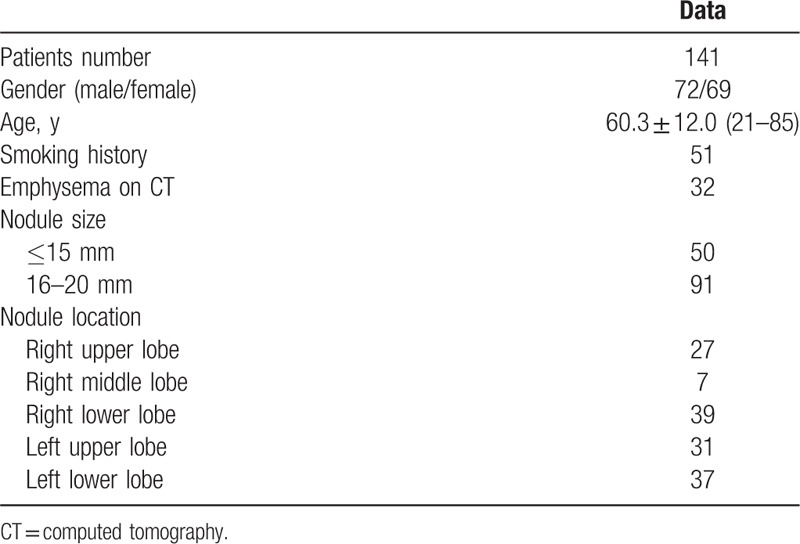
Baseline data of the 141 patients.

### 2.2 PCNB procedure

All the procedures were performed by 2 interventional radiologists and a pathologist. PCNB was guided by the 16-detector CT (Philips, Cleveland, OH). The tube voltage and current were 120 kilovolt and 150 milliampere second, respectively.

Patients were placed in prone, supine, or lateral decubitus positions according to the location of the target nodule. If the patient had multiple lung nodules, the target nodule was chosen as the largest nodule. If there was no appropriate puncture pathway for the largest nodule, the other nodule was chosen.

**Figure 1:**
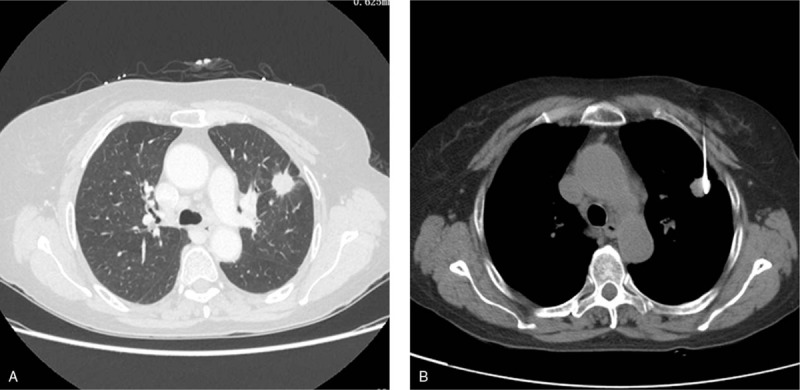
A 70-year-old female underwent CT-guided PCNB for small lung nodule (adenocarcinoma). A, CT scan demonstrated the small lung nodule at left upper lobe. B, The lung nodule was punctured for biopsy. CT = computed tomography, PCNB = percutaneous cutting needle biopsy.

The needle pathway and length from puncture site to lesion were evaluated by preoperative thoracic CT scan. The routine section thickness was 5 mm, if we could not find an appropriate puncture pathway under the 5 mm section thickness, the section thickness was exchanged to 2 mm. The needle pathway was selected to avoid bone, visible vessels, bullae, and fissures. The puncture site was chosen by the CT gantry laser lights and landmarks using a self-made radiopaque grid on the patient's skin. The local anaesthesia was performed with 5 mL 2% lidocaine. A 18 G cutting needle (Precisa, Roma, Italy, or Wego, Weihai, China) was punctured into the lung, and the repeat CT scan was performed to evaluated the site of the needle. When the needle tip touched the lesion, the specimen was obtained (Fig. 1). The obtained specimen was reviewed by the pathologist, if the specimen quantity was sufficient, the procedure was finished; otherwise, another specimen was required. The obtained specimen was placed into 10% formaldehyde for pathological examination.

After the PCNB procedure, chest CT was performed instantly to detect the pneumothorax or lung hemorrhage. All the patients were observed for 24 hours after PCNB.

### 2.3 Definitions and endpoints

Technical success of PCNB is defined as obtaining adequate tissue sampling on visual inspection.^[13]^ PCNB results were divided into the following diagnostic categories: malignancy; suspected malignancy; negative for malignancy; and invalid diagnosis (necrotic tissue or alveolus tissue). Malignancy and suspected malignancy were considered the positive results.^[14]^ Negative for malignancy was considered the negative results.^[14]^ The invalid diagnosis was considered neither positive nor negative.^[14]^ The final diagnosis of the target nodule was confirmed in 1 of the 4 ways: surgical resection; if pathological analysis of PCNB revealed malignancy or specific benign lesion (such as hamartoma, mycotic infection, or tuberculosis), these results were accepted as the final diagnosis;^[13,15]^ in the case of nonspecific benign lesion (such as chronic inflammation) or suspected malignancy, nodules were considered benign only when they decreased 20% or more in diameter or were stable in size for at least 2 years;^[13,15]^ if nonspecific benign lesion or suspected malignancy did not meet the third criterion or patient underwent anticancer treatment during the follow-up period, the final diagnosis was listed as nondiagnostic lesion. The PCNB positive result was considered true positive if the target nodule was proven as malignancy on final diagnosis. The PCNB negative result was considered true negative if the target nodule was proven as benign on final diagnosis. The invalid diagnosis on PCNB and nondiagnostic lesion on final diagnosis were not included for calculating the diagnostic accuracy.

The severity of lung hemorrhage was divided into 4 grades: grade 1 as needle tract hemorrhage 2 cm or less in width; grade 2 as hemorrhage more than 2 cm in width but sublobar; grade 3 as lobar hemorrhage or greater; hemothorax.^[16]^

The primary endpoint was diagnostic accuracy. The secondary endpoints included pneumothorax and lung hemorrhage.

### 2.4 Statistical analysis

Statistical analysis was performed by using SPSS 16.0 (SPSS Inc, Chicago, IL). Continuous variables were summarized as the mean or median. Numeric data were analyzed by using the *χ*^2^ test or Fisher exact probability test. The predictors of procedure-related complications were determined using univariate and multivariate logistic regression analysis. The covariates incorporated into the multivariate analysis were the variables with *P* < .1 on univariate analysis. A *P* value < .05 was considered statistically significant.

## 3 Results

### 3.1 Technical success

During the enrollment period, a total of 141 patients with 141 nodules were punctured. Technical success of CT-guided PCNB for small lung nodule was 100%. The details of PCNB procedure were demonstrated in Table 2.

**Table 2:**
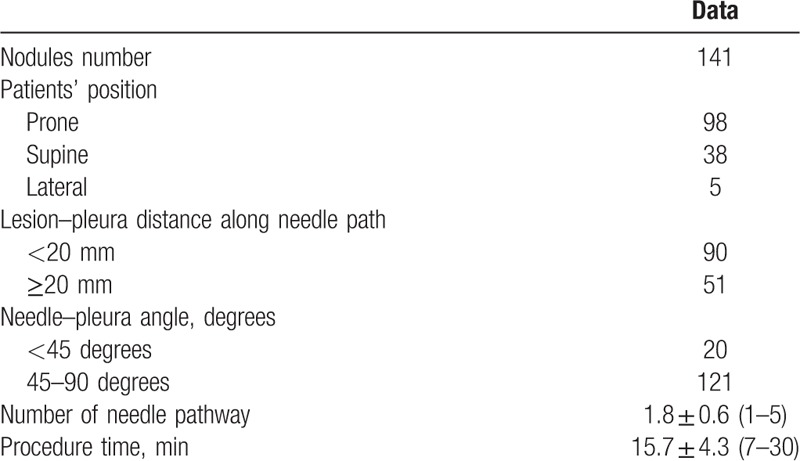
The details of lung biopsy procedure.

### 3.2 Diagnostic accuracy

**Figure 2:**
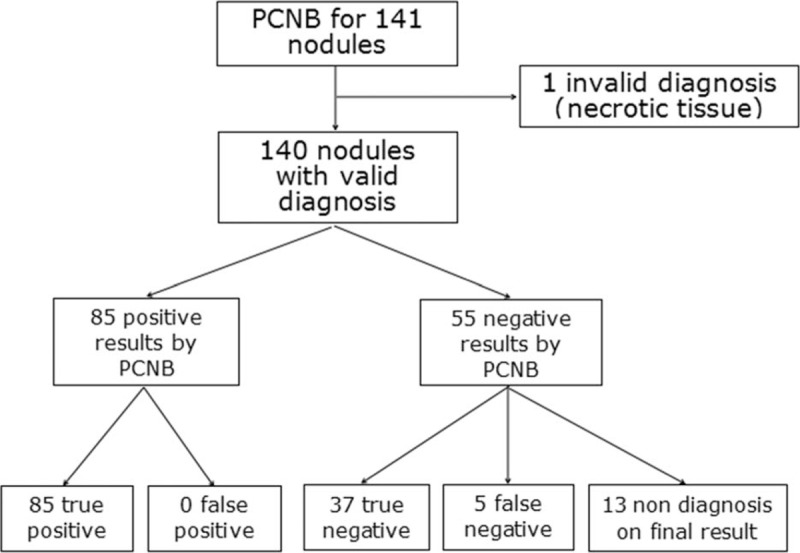
Flow diagram of PCNB and final results. PCNB = percutaneous cutting needle biopsy.

PCNB results included malignancy (n = 79), suspected malignancy (n = 6), specific benign lesion (n = 8), nonspecific benign lesion (n = 47), and invalid diagnosis (n = 1). The 79 malignant and 8 specific benign results were directly considered the final results. The 6 suspected malignant nodules were confirmed as adenocarcinoma by surgical resection. Among the 47 nonspecific benign nodules, 29 nodules were confirmed as benign lesions by follow-up (n = 21) or surgical resection (n = 8), 5 nodules were confirmed as malignancy by surgical resection (n = 3) or the second time PCNB (n = 2) due to the high carcino-embryonic antigen level or bone metastases on emission CT, and 13 nodules were nondiagnostic lesions (Fig. 2). The 1 invalid diagnostic nodule was nondiagnostic lesion at final diagnosis. The reasons of nondiagnostic lesion included lost of follow-up (n = 11) and anticancer treatment during the follow-up (n = 3). Therefore, 90 nodules were malignancy, 37 nodules were benign, and 14 nodules were nondiagnostic lesions at final diagnostic. The 90 malignant nodules included adenocarcinoma (n = 69), small cell lung cancer (n = 9), squamous carcinoma (n = 7), adenosquamous carcinoma (n = 2), neuroendocrine carcinoma (n = 2), and metastases (n = 1). The 14 nondiagnostic nodules were not included for calculating the diagnostic accuracy. Finally, the sensitivity, specificity, and overall diagnostic accuracy of PCNB for small lung nodule were 94.4% (85/90), 100% (37/37), and 96.1% (122/127), respectively. In addition, there were no significant difference in sensitivity, specificity, and overall diagnostic accuracy between nodules with different size (>15 vs ≤15 mm, Table 3).

**Table 3:**
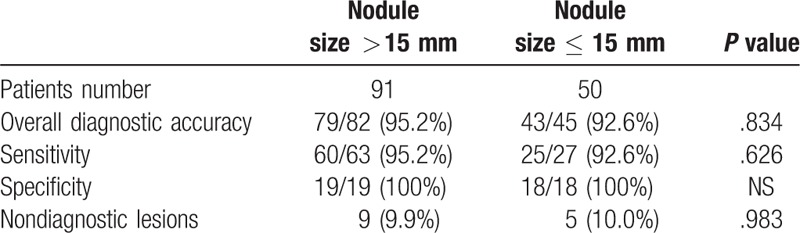
Comparison of diagnostic accuracy between subgroups.

### 3.3 Complications

Seventeen patients (12.1%) experienced pneumothorax after PCNB. Among the 17 patients, 14 patients experienced instant pneumothorax after PCNB and 3 patients experienced delayed pneumothorax. Among the 17 patients, 9 patients were managed by chest tube insertion, and 8 patients were managed by conservative treatment. At univariate and multivariate analyses, the risk factors of pneumothorax included nonprone position (*P* = .019) and longer procedure time (*P* = .018, Table 4).

**Table 4:**
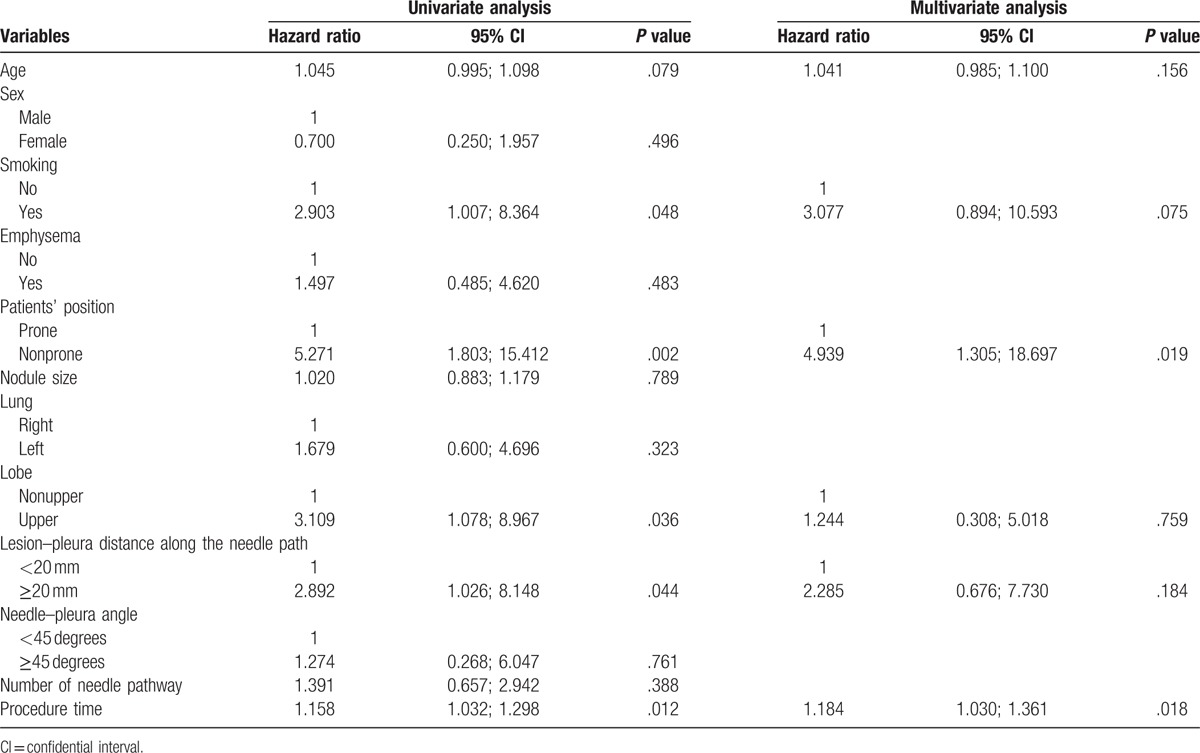
Univariate and multivariate logistic analyses of pneumothorax.

Forty-six patients (32.6%) experienced lung hemorrhage after PCNB. Among them, 21 patients experienced lung hemorrhage with grade 2 and 1 patient experienced lung hemorrhage with grade 3. These 46 patients were successfully managed by hemostasis. At univariate and multivariate analyses, the independent risk factor of lung hemorrhage (≥grade 2) was deeper lesion distance from pleura along needle path (*P* = .024).

## 4 Discussion

This study determined the feasibility and diagnostic accuracy of CT-guided PCNB for small lung nodules. The technical success rate is 100% and the overall diagnostic accuracy is 96.1%. These results may indicate that CT-guided PCNB is an effective method for diagnosing small lung nodules.

At present, the managements of lung nodule mainly included 3 methods. First, follow-up; second, lung biopsy; third, surgical resection.^[1]^ The lung nodules were considered benign according to follow-up only when they decreased 20% or more in diameter or were stable in size for at least 2 years.^[13,15]^ However, follow-up usually brings a huge mental press to patients. Although surgical resection is the gold standard for the diagnosis of lung nodule, it is an invasive procedure and may be associated with morbidity and even mortality.^[17]^ Furthermore, surgical resection is not suit for some patients with multiple lung nodules or with distant metastases.

CT-guided lung biopsy has been widely used in pathology diagnosis for lung lesions.^[9–12]^ Previous studies of FNAB for small lung nodules (≤20 mm) demonstrated that diagnostic accuracy was only 64.6% to 77.2%.^[18,19]^ In this present study, the diagnostic accuracy of PCNB for small lung nodules is 96.1%. This rate is higher than the diagnostic accuracy of FNAB for small lung nodule and is comparable to the previous study of CT-guided PCNB for small lung nodules.^[9]^ Ocak et al^[12]^ compared the diagnostic accuracy between PCNB and FNAB for lung lesions, although there was no significant difference in overall diagnostic accuracy between 2 groups, PCNB provided a well-defined cancer type/subtype. In addition, PCNB specimens can provide adequate tissues for molecular testing, which can guide the treatment strategy of lung cancer.^[10]^

Tian et al ^[10]^ discovered that smaller size of lung nodule was associated with higher diagnostic failure rate. Puncture of a small lung nodule usually presents a high technical difficulty.^[10]^ In this present study, the technical success of CT-guided PCNB for small lung nodules was 100%. In addition, there were no significant difference in diagnostic accuracy between nodules with different size.

In recent years, the CT fluoroscopy technique has been widely used in lung biopsy.^[14,20–22]^ Compared with the conventional CT-guided lung biopsy, CT fluoroscopy guidance allows the physician to continuously monitor the needle as it progresses toward the target lesion. Kim et al^[14]^ compared the diagnostic accuracy and safety between CT fluoroscopy-guided lung biopsy and conventional CT-guided lung biopsy. The results demonstrated that there was no significant difference in diagnostic accuracy between 2 groups but a lower complication rate in CT fluoroscopy-guided group (*P* = .012). However, CT fluoroscopy-guided lung biopsy requires real-time monitoring; therefore, radiation exposure to both patient and doctor was significantly higher in fluoroscopy-guided group.

Pneumothorax is a common lung biopsy-related complication. In this present study, the incidence of pneumothorax is 12.1%, which is comparable to that in the previous study of CT-guided PCNB for small lung nodule.^[9]^ In the Ocak study,^[12]^ although the rate of pneumothorax was significant higher with PCNB versus FNAB (31% vs 19%, *P* = .004), the chest tube insertion rates were similar (10% vs 11%). This result may indicate that fine-needle could not effectively decrease the rate of pneumothorax. In this present study, the risk factors of pneumothorax after PCNB included nonprone position and longer procedure time. The back muscle is usually thicker than the thoracic muscle; therefore, the needle is more stable when it is punctured from back. It may explain that nonprone position was associated with a high risk of pneumothorax after PCNB. Longer procedure time may cause a greater chance of tearing the pleura and lung tissue as patient's breath during the biopsy procedure.^[23]^ Thus, longer procedure time is associated with a high risk of pneumothorax is reasonable.

Lung hemorrhage (≥grade 2) occurred in 22 (15.6%) patients in this present study. Grade 2 or higher lung hemorrhage is considered higher grade hemorrhage.^[16]^ The independent risk factor of lung hemorrhage (≥grade 2) after PCNB was deeper lesion distance from pleura along needle path in this present study. This result is comparable to previous studies about lung hemorrhage after PCNB.^[9,23]^ Although lung hemorrhage is a potentially life-threatening complication, all the patients with lung hemorrhage in this study were successfully managed by conservative treatment.

This study has some limitations. First, this is a retrospective review, and therefore the selection bias does exist. Second, 14 of 141 nodules (9.9%) were considered nondiagnosis. Although the nondiagnostic lesions were also reported in previous studies of lung biopsy,^[13–15]^ they definitely influenced the diagnostic accuracy. Third, there is no control group. Therefore, we have no means of comparing this approach to CT fluoroscopy-guided or C-arm cone-beam CT guided PCNB for small lung nodule. Further perspective controlled trial should be performed.

In conclusion, although further clinical trials are needed, our study indicates that CT-guided PCNB can provide a high diagnostic accuracy for small lung nodule (≤20 mm) with acceptable complications.
